# Response to the letter to the editor regarding “prediction accuracy ofcochlear implant electrode insertion using the OTOPLAN software”

**DOI:** 10.1007/s00405-025-09638-9

**Published:** 2025-08-25

**Authors:** Caterina Vazzana, Marten Geisen, Uwe Baumann, Dennis Sakmen, Esther Knörle, Timo Stöver, Silke Helbig

**Affiliations:** 1 Department of Otorhinolaryngology, Head and Neck Surgery, Goethe University Frankfurt, University Hospital, Theodor-Stern-Kai 7, D-60590 Frankfurt, Germany; 2 Department of Audiology & Audiological Acoustic, Goethe University Frankfurt, University Hospital, Frankfurt, Germany

Dear Dr. Khan,

Thank you for your interest in our article and your thoughtful comments. We appreciate the opportunity to clarify several methodological points you raised. Many of these aspects were already discussed in our manuscript, but we are happy to reiterate them here for clarity.

1. Inter-Rater Variability. You rightly highlight inter-rater variability as an important consideration when using imaging software. This issue has been thoroughly studied for OTOPLAN. As cited in our article, studies by Paouris et al. and Canfarotta et al. demonstrate excellent inter- and intra-rater reliability for cochlear measurements, insertion depth, and frequency mapping. To ensure procedural consistency and minimize variability, all measurements in our study were performed by a single experienced surgeon. This approach aligns with best practices to reduce intra-study variance.
2. Imaging Modality: CT and CBCT. As described in our methods and limitations sections, we employed CT preoperatively and CBCT postoperatively, reflecting standard clinical workflows at our center and many others. Although CBCT is more prone to motion artifacts, it remains an accepted postoperative evaluation modality due to its lower radiation dose. We explicitly discussed this trade-off, noting that while deviations between CT and CBCT were statistically significant, they were minimal and not clinically relevant in our context.
3. Gender-Based Differences in Cochlear Duct Length. Our study identified a statistically significant difference in cochlear duct length (CDL) between male and female patients, consistent with prior literature. This finding was detailed in the results and addressed in the conclusion. Importantly, OTOPLAN provides individualized measurements, and electrode choice is based on these patient-specific data rather than sex alone. However, as we noted, in the absence of preoperative imaging, knowledge of sex-based anatomical differences can help guide default electrode selection: “Female cochlea size was found to be statistically significantly smaller than male cochlea. In the absence of preoperative measurements, this information can inform the surgeon’s choice of a sex-specific standard electrode.”
4. Limitation to MED-EL Electrode Arrays. We acknowledged that OTOPLAN is currently optimized for MED-EL electrode designs. Nevertheless, key anatomical measurements such as CDL and angular insertion depth have general relevance and support implant planning across different systems. Notably, MED-EL is currently the only manufacturer offering lateral wall electrodes longer than 24 mm, up to 34 mm, making preoperative CDL measurement particularly important in this context. We agree Response to Letter to Editor that expanding software calibration to other manufacturers would enhance clinical utility and generalizability.

We thank you again for your detailed feedback. The methodological considerations you raised are valid but were addressed transparently in our study. We hope this clarification reinforces the rigor of our work and contributes to the ongoing discussion on the value of preoperative planning tools in cochlear implantation.

Sincerely,

Silke Helbig and Caterina Vazzana

On behalf of all authors

